# Morphological development and cytochrome *c* oxidase activity in *Streptomyces lividans* are dependent on the action of a copper bound Sco protein

**DOI:** 10.1098/rsob.120163

**Published:** 2013-01

**Authors:** Katie L. I. M. Blundell, Michael T. Wilson, Dimitri A. Svistunenko, Erik Vijgenboom, Jonathan A. R. Worrall

**Affiliations:** 1 School of Biological Sciences, University of Essex, Wivenhoe Park, Colchester CO4 3SQ, UK; 2 Molecular Biotechnology, Institute of Biology Leiden, Sylvius Laboratory, Leiden University, PO Box 9505, 2300RA Leiden, The Netherlands

**Keywords:** *Streptomyces*, copper-metallochaperone, cytochrome *c* oxidase, Sco protein, morphological development, thiol-disulphide reductase activity

## Abstract

Copper has an important role in the life cycle of many streptomycetes, stimulating the developmental switch between vegetative mycelium and aerial hyphae concomitant with the production of antibiotics. In streptomycetes, a gene encoding for a putative Sco-like protein has been identified and is part of an operon that contains two other genes predicted to handle cellular copper. We report on the Sco-like protein from *Streptomyces lividans* (Sco^Sl^) and present a series of experiments that firmly establish a role for Sco^Sl^ as a copper metallochaperone as opposed to a role as a thiol-disulphide reductase that has been assigned to other bacterial Sco proteins. Under low copper concentrations, a Δ*sco* mutant in *S. lividans* displays two phenotypes; the development switch between vegetative mycelium and aerial hyphae stalls and cytochrome *c* oxidase (CcO) activity is significantly decreased. At elevated copper levels, the development and CcO activity in the Δ*sco* mutant are restored to wild-type levels and are thus independent of Sco^Sl^. A CcO knockout reveals that morphological development is independent of CcO activity leading us to suggest that Sco^Sl^ has at least two targets in *S. lividans*. We establish that one Sco^Sl^ target is the dinuclear Cu_A_ domain of CcO and it is the cupric form of Sco^Sl^ that is functionally active. The mechanism of cupric ion capture by Sco^Sl^ has been investigated, and an important role for a conserved His residue is identified.

## 2. Introduction

Streptomycetes are soil-dwelling Gram-positive bacteria best known for their ability to produce a bewildering array of secondary metabolites that have antibiotic, antimicrobial and anti-cancer properties [1]. The onset of chemical differentiation (antibiotic production) coincides with the onset of morphological development from a vegetative growth to a reproductive growth phase beginning with aerial mycelium followed by cell division and sporulation. The bioavailability of copper (Cu) has been shown in certain *Streptomyces* strains to stimulate morphological development and antibiotic production [2–5]. This dependence is restricted to the reproductive growth phase (aerial mycelium and spores), whereas vegetative growth proceeds under strongly Cu-limiting conditions. The Cu proteome of *Streptomyces coelicolor* has been reported based on *in silico* methods and contains a large number of cuproproteins and cupro enzymes [6,7]. The stimulatory effect of Cu in development therefore suggests that some Cu-dependent biochemical functions correlate with the complex life cycle of streptomycetes and that cuproproteins and cuproenzymes must play a part. However, their identification and role in development have yet to be fully elucidated [7].

**Figure 1.**
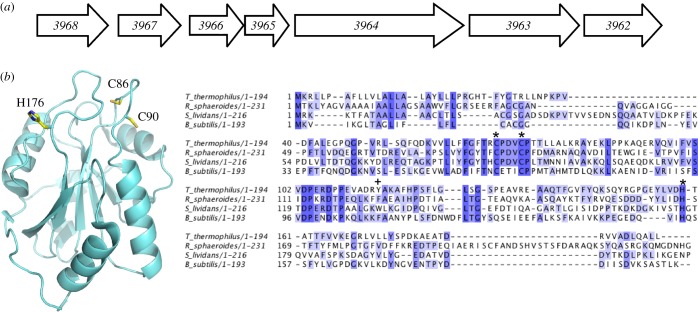
The *sco* operon, X-ray crystal structure of apo-Sco^Bs^ and multiple sequence alignment of bacterial Sco proteins for which biochemical or structural data have been reported. (*a*) Schematic of the unidirectional *sco* operon (*3966*, *3965* and *3964*), including two upstream and two downstream genes found in *Streptomyces coelicolor*. The coding sequence numbers assigned in the *S. coelicolor* genome are indicated with the respective annotations obtained from the StrepDB; *3968* (integral membrane protein), *3967* (hypothetical membrane protein), *3966* (Sco/SenC/PrrC-like protein), *3965* (conserved hypothetical protein), *3964* (integral membrane putative Cu transport protein with Cu binding CopC-like and CopD-like domains), *3963* (hypothetical protein) *3962* (prephenate dehydratase involved in Phe biosynthesis). (*b*) Cartoon representation of the X-ray crystal structure of apo-Sco^Bs^ (pdb 1XZO) [10]. The location of the His and Cys residues considered to be involved in Cu(II) coordination are labelled with *S. lividans* numbering. ClustalW2 sequence alignments [11] of bacterial Sco proteins with completely and partially conserved residues boxed from dark blue to light blue, respectively. The residues involved in Cu(II) coordination are indicated (asterisk) and a plus symbol indicates the position of Trp-132 for *S. lividans*. The UniprotKB NCBI accession numbers are gi |257157333| (*Bacillus subtilis*), gi |77386477| (*Rhodobacter sphaeroides*), gi |198443051| (*Thermus thermophilus*).

*Streptomyces lividans* has an even more pronounced dependence on Cu for development than *S. coelicolor*. The former shares a high level of sequence identity and genome organization with *S. coelicolor* but the annotated genome has not yet been reported. Therefore, the gene numbering of the annotated *S. coelicolor* genome database is used in this study in keeping with previous studies of cuproproteins in *S. lividans* [8,9]. A recent study has highlighted that in the five sequenced and annotated genomes of streptomycetes a conserved unidirectional gene cluster is present that includes three genes (*3966*, *3965*, *3964*) predicted to encode cuproproteins (figure 1) [4]. In *S. coelicolor*, experimental evidence suggests that this cluster has a central role in the utilization of Cu and may therefore have a complex role in the Cu-dependent stimulation of development [4]. The operon has been named the *sco* operon owing to the presence of a gene encoding for a protein with a CXXXC motif and sequence homology with the Sco/SenC/PrrC protein family. Synthesis of cytochrome *c* oxidase (Sco) proteins is present in both eukaryotes and prokaryotes, and was first identified, along with other cytochrome *c* oxidase (CcO) assembly factors, in the mitochondria of yeast and then subsequently in humans where a role as a Cu(I)-metallochaperone in co-factoring the mixed valance di-nuclear Cu_A_ site of the *aa*_3_-type CcO of the mitochondrial electron transport chain was first proposed [12–14].

The most extensively studied bacterial Sco protein is that from *Bacillus subtilis* (Sco^Bs^). This protein has been reported to bind both Cu(I) and Cu(II) *in vitro,* and a genetic knockout of Sco^Bs^ leads to a marked phenotype on the activity of the *aa*_3_-type CcO and organism viability [15] suggesting a role in Cu_A_ site maturation in this Gram-positive bacteria. An alternative role as a thiol-disulphide reductase has been proposed for bacterial Sco proteins [10,16,17]. Evidence for this comes from an *in vitro* NMR study using the periplasmic Sco protein form *Thermus thermophilus* (Sco^Tt^), which was found to be incapable of delivering Cu to the Cu_A_ site of CcO [18]. Instead, a role in keeping the Cys residues of the Cu_A_ site in the reduced form to facilitate metallation of the Cu_A_ site by a newly identified periplasmic Cu(I) chaperone, PCu_A_C (periplasmic Cu_A_ chaperone) [18], was proposed. PCu_A_C is not present in all bacteria and is absent in *B. subtilis*, but in *S. lividans* the *3965* gene, part of the *sco* operon (figure 1*a*), is predicted to encode a PCu_A_C-like protein [7]. The structures of Sco proteins from yeast [19], human [20,21], *B. subtilis* [10,22] and *T. thermophilus* [18] all have a fold very similar to the thioredoxin (Trx) superfamily (figure 1*b*), and, importantly, the CXXXC motif implicated in Cu binding (figure 1*b*) superimposes on the CXXC redox motif of Trx making a redox role for Sco proteins an attractive proposition. Subsequent studies have reported thiol-disulphide reductase activity of Sco^Bs^ and PrrC (a Sco-like protein found in *Rhodobacter sphaeroides*) [23,24].

A consensus functional role of bacterial Sco proteins has not therefore been reached and it may be that variation exists across species [17]. In this study, we have focused our attention on the Sco-like protein identified in *S. lividans* (Sco^Sl^), in which the gene (*3966*) is located in the so-called *sco* operon (figure 1*a*) [4]. We report herein on the properties of an N-terminal-truncated form of the *3966* gene product upon addition of cupric ions and assess the redox and thiol-disulphide reductase activity of the CXXXC motif in the absence of Cu. Parallel *in vivo* studies have been performed and imply a critical role for Sco^Sl^ both in the development switch from vegetative mycelium to aerial hyphae and in the activity of the *aa*_3_-type CcO at low [Cu]. The development switch is found to be independent of CcO activity, and we present evidence to suggest that a second Sco^Sl^ target, requiring Cu for function, is essential for morphological development in *S. lividans* under low Cu levels. Mechanistic features of Cu(II) loading to Sco^Sl^ and the H176A mutation have been studied, which together with the *in vivo* data leads to new insights into the functional role of Sco^Sl^ in *S. lividans*.

## 3. Material and methods

### 3.1. Generation of the Sco and Cox mutants of *Streptomyces lividans* 1326

The agar media soya flower mannitol (SFM), R5 (complex medium) and Difco nutrient agar (DNA) were prepared according to Kieser *et al.* [25]. Antibiotics were used in the following final concentrations: apramycin (50 μg μl^−1^) and thiostrepton (5–20 μg μl^−1^). Agar plates were incubated at 30°C, and spore stocks were obtained from cultures grown on SFM plates and stored in 20 per cent glycerol at −20°C. The *3966* gene encoding for Sco^Sl^ was deleted in *S. lividans* 1326 (*S. lividans* 66, stock number 1326 from the John Innes Centre) in a two-step process using the CRE-lox system [26]. First, the gene (nt + 33 to 490) was replaced by homologous recombination with an apramycin-resistance cassette flanked by loxP sites. For this purpose, the upstream flanking region of *3966* (from −2160 to +32) and the downstream flanking region (from +491 to 2471) were amplified from genomic DNA by PCR introducing *Eco*RI, *Xba*I and *Xba*I, *Hind*III sites, respectively, for cloning purposes. These two fragments and the apramycin-resistance cassette flanked by loxP sites were cloned in the delivery vector pWHM3 that is unstable in *Streptomyces* [27]. Following protoplast transformation, recombinants that were apramycin-resistant but had lost the vector (thiostrepton-resistance) were isolated. Second, an unstable, plasmid-encoding Cre recombinase was introduced [26]. The Cre recombinase allowed for the excision of the apramycin-resistance cassette on the lox sites. The resulting strain, Δ*sco*, has lost most of the coding sequence of Sco^Sl^ and has only a 61 nt scar, including two *Xba*I sites left in the genome. The Δ*sco* strain was analysed by PCR to confirm the loss of *3966* and vector sequences. The *cox*::Apra mutant was isolated with a similar protocol replacing the open reading frames of *2155* and *2156* with the apramycin-resistance cassette. The replacement was confirmed by PCR.

### 3.2. Plasmid construction for complementation

For complementation/over-expression of Sco^Sl^ in wild-type (wt) 1326 and Δ*sco* strains of *S. lividans*, the *3966* gene including 747 nt upstream was cloned in pHJL401 as an *Eco*RI–*Bam*HI fragment generated by PCR with genomic DNA as template. The 747 nt upstream contains the entire promoter region of *3966* as determined by promoter probing with the *redD* gene as reporter [28]. The Sco^Sl^ H176A mutation was introduced in this construct by fusion PCR of two fragments with a 24 nt overlap. The inserts of all plasmids used in this study were sequenced on both strands. Introduction of the plasmids in *S. lividans* was carried out by protoplast transformation according to standard protocols.

### 3.3. *In vivo* cytochrome *c* oxidase assay

The *in vivo* CcO activity was visualized with *N,N,N′,N′*-tetramethyl-*p*-phenylenediamine (TMPD) as substrate essentially according to references [29,30]. The wt and mutant strains were spotted on DNA plates (10 μl containing 1000 spores) and incubated for 24–30 h at 30°C. Spots were overlaid with 10 ml of a 25 mM phosphate buffer pH 7.4 solution containing 20 per cent ethanol, 0.6 per cent agarose, 1 per cent sodium deoxycholate and 10 mg TMPD. CcO activity is detected by the appearance of the blue colour of the oxidized product (indophenol blue) of the TMPD substrate and was recorded by taking digital images every 30 s for 5–10 min.

### 3.4. Cloning and site-directed mutagenesis of Sco^Sl^ for over-expression in *Escherichia coli*

An N-terminal-truncated version of Sco^Sl^ (*3966*), with nucleotides encoding amino acid residues 1–23 deleted, was amplified from *S. lividans* DNA and ligated into the *Nde*I and *Bam*H restriction sites of a pET28a vector (Novagen) to create an N-terminal His_6_-tagged construct for over-expression in *Escherichia coli*. The Quikchange (Stratagene) site-directed mutagenesis method was used to create the H176A mutation in the N-terminal-truncated Sco^Sl^. The following mutagenic primers were used, with the nucleotides changed to create the mutation in lower case: 5′-GATCGTCTCGACCgcCGGCACCCAGGTCGTC-3′ and 5′-CGACCTGGGTGCCGgcGGTCGAGACGATC-3′.

### 3.5. Over-expression and purification of Sco^Sl^ and the H176A mutation

Sco^Sl^ and H176A were over-expressed in *E. coli* strain BL21 (DE3) starting from overnight pre-cultures (2 ml 2xYT, 2 μl Kan (50 mg ml^−1^), 37°C) that were subsequently used to inoculate 750 ml of medium in 2 l flasks. At an OD_600_ of 0.6, 1 M isopropyl β-d-1-thiogalactopyranoside solution (Melford) was added to give a final concentration of 1 mM and the temperature decreased to 25°C. Cells were harvested after 16 h at 4000 r.p.m. and lysed using an EmulsiFlex-C5 cell disrupter (Avestin) followed by centrifugation at 18 000 r.p.m. for 20 min at 4°C. The clarified supernatant was loaded to a 5 ml Ni^2+^–NTA Sepharose column (GE Healthcare) equilibrated with buffer A (50 mM Tris/HCl, 500 mM NaCl, 20 mM imidazole, pH 8.0) and eluted with a linear imidazole gradient using buffer B (buffer A with 500 mM imidazole). A single peak at approximately 25 per cent buffer B was eluted from the column, and fractions were pooled and dialysed overnight at 4°C against buffer C (50 mM Tris/HCl, 150 mM NaCl, 1 mM EDTA, pH 8.0). Following dialysis, the N-terminal His_6_-tag was removed by incubating the protein at room temperature overnight in the presence of 125 U of thrombin (Sigma). The protein/thrombin mixture was reapplied to the Ni^2+^–NTA Sepharose column (GE Healthcare) and the flow-through collected and concentrated at 4°C using a centrifugal concentrator (Vivaspin) with a 5 kDa cut-off. Concentrated protein was loaded to a G75 Sephadex column (GE Healthcare) equilibrated with buffer D (buffer C with 2 mM dithiothreitol (DTT)). Fractions eluting in the major peak of the G75 column were analysed by 15 per cent SDS–PAGE and those deemed of good purity were concentrated and stored at −20°C until required. Electrospray ionization–mass spectrometry (ESI–MS) using a Micromass Quattro Ultima triple quadrupole instrument operating in the positive ion detection mode was used to determine the mass of the purified samples. Proteins were desalted and exchanged into 1 M ammonium acetate followed by a 1 : 20 dilution with a 50 per cent methanol and 1 per cent formic acid solution.

### 3.6. Preparation of apo- and Cu(II)-Sco^Sl^ for circular dichroism spectroscopy

Apo-proteins were reduced in an anaerobic chamber (DW Scientific, [O_2_] less than 2 ppm) with 10 mM DTT and desalted (twice) using a PD-10 column (GE Healthcare) into the desired DTT-free buffer. Concentrations of apo-proteins were determined by UV–vis spectroscopy (Varian Cary 50 UV–vis spectrophotometer) using an extinction coefficient of 14 565 M^−1^ cm^−1^ at 280 nm [31]. Cu(II)-loaded samples were prepared in 100 mM sodium phosphate, 50 mM NaCl, pH 7.5 starting from reduced apo-Sco^Sl^ samples incubated with an excess of Cu(II)SO_4_ for 30 min. Unbound Cu(II) was removed by desalting (PD-10 column) and concentrating at 4°C using a 5 kDa cut-off centrifugal concentrator (Vivaspin). Samples for circular dichroism (CD) analysis were exchanged into 10 mM KPi, 50 mM KF. Far-UV CD spectra were recorded between 260 and 175 nm and visible spectra between 600 and 300 nm at 20°C on a Chirascan CD spectrophotometer (Applied Photophysics, Leatherhead, UK) equipped with a thermostatic cell holder controlled with a Peltier system. CD spectra were analysed using DichroWeb [32,33] with the programs CDSSTR [34–36] and Contin-LL [37] and the databases 4, 7 [36,38] and SP175 [39].

### 3.7. Electron paramagnetic resonance spectroscopy

Wilmad SQ electron paramagnetic resonance (EPR) tubes (Wilmad Glass, Buena, NJ, USA) were used and filled with 200 μl of protein solutions (100 mM sodium phosphate, 50 mM NaCl pH 7.5) treated with a sub-stoichiometric amount of Cu(II)SO_4_ (50 μM protein + 45 μM Cu(II)SO_4_) and frozen in methanol kept on dry ice. EPR spectra were measured at 10 K on a Bruker EMX EPR spectrometer (X-band) at a modulation frequency of 100 kHz. A spherical high-quality Bruker resonator SP9703 and an Oxford Instruments liquid helium system were used to measure the low-temperature EPR spectra. The EPR spectra of the blank samples (frozen water) were subtracted from the EPR spectra of the protein samples to eliminate the baseline caused by the resonator's walls, quartz insert or quartz EPR tube. The baseline was corrected by subtraction of a polynomial line drawn through a set of points randomly chosen on the baseline.

### 3.8. Cu(II) binding to apo-Sco^Sl^ monitored by UV–vis and fluorescence spectroscopy

A stock solution of 100 mM Cu(II)SO_4_ was prepared anaerobically and diluted as required. Reduced apo-Sco^Sl^ proteins were prepared anaerobically in 100 mM sodium phosphate, 50 mM NaCl, pH 7.5. Titration of a Cu(II) solution of known concentration using a gastight syringe (Hamilton) into 100 μM of reduced apo-protein in an anaerobic quartz cuvette (Hellma) was monitored at 20°C in the UV–vis spectrum. Fluorescence spectroscopy was carried out on an LS 50B fluorimeter (Perkin Elmer). A Trp fluorescence emission spectrum was collected between 300 and 400 nm with excitation at 280 nm. The excitation slit and the emission slit were set at 5 nm, and the change in emission at approximately 330 nm was monitored upon titration of aliquots of a Cu(II) solution into a 5 µM protein solution.

### 3.9. Stopped-flow kinetics

Kinetic experiments were carried out using an SX20 stopped-flow spectrophotometer (Applied Photophysics) thermostatted at 20°C with a Peltier system. A stock of 100 mM Cu(II)SO_4_ and reduced apo-proteins (20 mM sodium phosphate 50 mM NaCl, pH 7.5) were prepared. Time courses were taken at 360 and 380 nm with various Cu(II) concentrations (20–1000 µM before mixing) and 10 µM protein with the transients fitted to a two-step exponential function. A point-to-point accumulation was carried out over 320–420 nm with a step of 5 nm using 30 µM protein and 100 µM Cu(II) before mixing. The resulting kinetic data were analysed using the program ‘ProK’ (Applied Photophysics). This program was also used to construct spectra of intermediates using a simple sequential model (e.g. a > b > c). Analysis by singular value decomposition was used to determine the minimum number of species required to reconstruct the original dataset.

### 3.10. Determination of the mid-point redox potential of apo-proteins

Apo-Sco^Sl^ (2.5 μM) and the H176A mutant (2.5 μM) in 100 mM sodium phosphate, 50 mM NaCl, pH 7.5 were oxidized by the addition of 1 mM oxidized DTT (Sigma). The oxidized proteins were titrated with a stock solution of 10 mM reduced DTT (Melford), allowing 10 min for the protein to equilibrate to each new potential. The transition from oxidized to reduced protein was monitored by the increase in Trp fluorescence emission at approximately 330 nm (excitation at 280 nm) measured at 25°C with 5 nm excitation and emission slits. Intensity was corrected for dilution effects. A mid-point reduction potential (*E*_m_) was determined by calculating the fraction of Sco^Sl^ reduced (*f*_r_) at each point in the titration and plotting this as a function of the system potential. The data were then fit to the Nernst equation (3.1)3.1
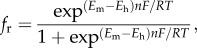
in which *E*_m_ and *n*, the number of electrons, were allowed to float. *F* is the Faraday constant, *R* is the gas constant, *T* is the temperature and *E*_h_ is the standard reduction potential couple for DTT, which was taken to be −330 mV at pH 7 and 25°C.

### 3.11. Thiol-reductase activity assay

The turbidimetric insulin assay as outlined by Holmgren [40] was used to monitor thiol-reductase activity. *E. coli* thioredoxin (Trx) and human insulin were purchased from Sigma and reaction mixtures containing 3 μM of protein and 2.5 mM DTT were incubated in K_2_HPO_4_, pH 8 followed by the addition of insulin to a final concentration of 0.17 mM. The absorbance change at 650 nm caused by the precipitation of reduced insulin chains was monitored using a Cary 50 spectrophotometer every 60 s for up to 2 h. Controls were carried out with Trx in the absence of DTT, bovine serum albumin (BSA) in the presence of DTT and insulin with DTT.

## 4. Results

### 4.1. Sco^Sl^ is over-expressed, folded and has two reactive Cys residues

**Figure 2.**
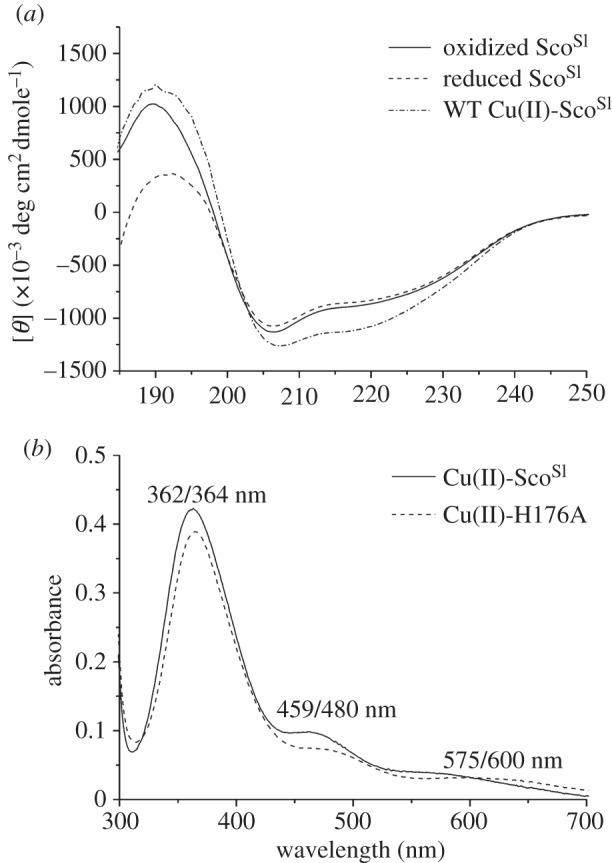
CD and visible absorption spectra of Sco^Sl^ and the H176A mutant. (*a*) Far-UV CD spectra of apo-oxidized (disulphide), apo-reduced (free-thiol) and Cu(II)-loaded Sco^Sl^. (*b*) Visible absorbance spectra of Cu(II)-loaded Sco^Sl^ and the H176A mutant. All spectra were recorded at 20°C, with protein concentrations ranging between 20 and 100 μM.

An N-terminal His_6_-tag fusion construct in which the first 23 amino acids of Sco^Sl^ were absent was prepared. The design of this construct was based on bioinformatics analysis of the Sco^Sl^ amino acid sequence in which an N-terminal transmembrane helix or a signal sequence (residues 1–21) for secretion with putative lipidation of Cys-22, which subsequently becomes the N-terminal amino acid upon cleavage by signal peptidase II, was predicted. The purified protein had a mass of 21 265.8 (1.1) Da (predicted mass, 21 264 Da) determined by denaturing ESI–MS. This mass corroborated that the N-terminal fused His_6_-tag had been successfully removed by thrombin treatment during the purification stages (mass with His_6_-tag 23 147.9 Da). The visible region of the absorption spectra was featureless with a prominent peak in the UV region at 280 nm. Anaerobic treatment of the purified Sco^Sl^ with DTT and subsequent removal by desalting under anaerobic conditions yielded a protein with accessible thiol groups as determined by the reduction of DTNB (5,5′-dithiobis-(2-nitrobenzoic acid) [41]. An average thiol : protein ratio of 1.6 : 1 was consistently determined, which is close to the expected ratio of 2 : 1 based on the presence of two Cys residues in the CXXXC motif (figure 1*b*). The far-UV CD spectra of reduced and oxidized Sco^Sl^ are shown in figure 2*a* and are typical for a mixed secondary structure protein whereby the α-helical segments are dominant with some distortion in the ‘classical’ α-helical CD shape resulting from β-strand segments. Secondary structure content of these two Sco^Sl^ forms was determined using DichroWeb and is reported in table 1.

**Table 1.** Output from DichroWeb of average secondary structure content in the different forms of Sco^Sl^ used in this study.

protein	*α*-helix (%)	*β*-sheet (% )	turn (%)	unordered (%)
oxidized Sco^Sl^	27.3	17.1	20.9	34.7
reduced Sco^Sl^	35.0	18.7	16.2	30.0
Cu(II)-Sco^Sl^	34.6	17.3	18.0	30.0
oxidized H176A	29.2	15.1	19.9	35.8
reduced H176A	27.1	18.6	20.9	33.4
Cu(II)-H176A	26.1	21.0	21.7	31.2

### 4.2. Reduced apo-Sco^Sl^ binds Cu(II)

**Figure 3.**
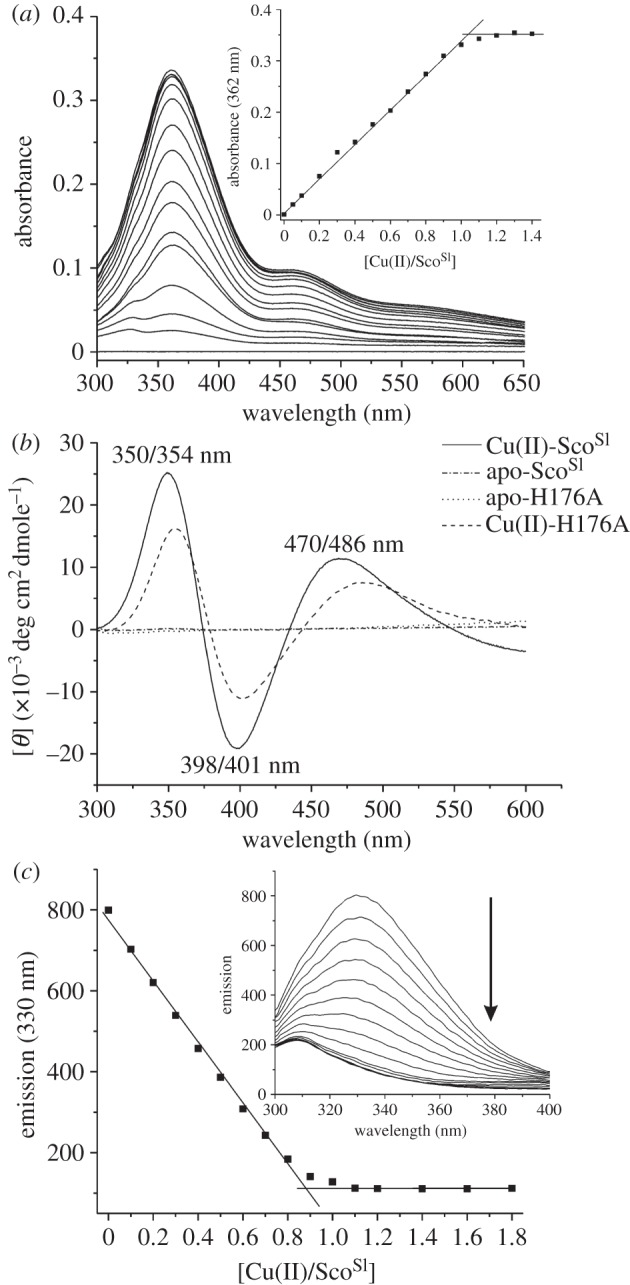
Cu(II) binding to reduced apo-Sco^Sl^. (*a*) Changes in the UV–vis spectrum (pH 7.5, 20°C) of reduced apo-Sco^Sl^ (110 μM) upon stepwise addition of 10 μM Cu(II)SO_4_. Inset: the change in absorbance at 362 nm plotted as a function of [Cu(II)]/[Sco^Sl^]. (*b*) Visible CD spectra of Cu(II)-loaded and apo-reduced Sco^Sl^ and the H176A mutant. (*c*) Inset: changes in the Trp emission spectrum (pH 7.5, 20°C) of reduced apo-Sco^Sl^ (5 μM) upon titration with 0.5 μM of Cu(II)SO_4_ with the emission at 330 nm plotted as a function of [Cu(II)]/[Sco^Sl^]. The arrow indicates the direction of the emission change. The stoichiometry of the reaction in both (*a*) and (*c*) is indicated by the intersection of the two lines.

To determine whether Sco^Sl^ has affinity for Cu(II), reduced apo-Sco^Sl^ was titrated with a Cu(II) solution. A number of distinct absorption bands in the UV–vis spectrum appear (figure 2*b*). The *λ*_max_ are similar, but not identical, to those reported for Cu(II)-Sco^Bs^ (354, 450 and 545 nm) and have been attributed to arise from S(Cys)–Cu(II) charge transfer [42]. The absorbance increase at 362 nm plotted as a function of [Cu(II)]/[Sco^Sl^] reveals a steep linear transition with a break point at approximately 1 equivalent of Cu(II) (figure 3*a*, inset), indicating a 1 : 1 binding stoichiometry with a strong affinity for Cu(II). In the presence of excess EDTA (up to 50-fold), the intensity of the absorption peaks in the UV–vis spectra of Cu(II)-Sco^Sl^ remained unchanged over many days. The far-UV CD spectrum of Cu(II)-Sco^Sl^ is similar to the apo-reduced or -oxidized forms (figure 2*a*) and thus consistent with no gross secondary structural change upon Cu(II) binding (table 1). The visible CD spectrum for the Cu(II)-Sco^Sl^ complex is shown in figure 3*b* and has a number of peaks and troughs that probably arise from S(Cys)–Cu(II) charge transfer. A single Trp residue (W132) is present in Sco^Sl^ (figure 1*b*). Excitation of reduced apo-Sco^Sl^ at 280 nm results in an emission spectrum with a peak maximum at approximately 330 nm, which upon addition of Cu(II) is quenched (figure 3*c*, inset). A plot of the emission intensity at 330 nm versus [Cu(II)]/[Sco^Sl^] indicates a 1 : 1 binding stoichiometry (figure 3*c*) consistent with the data from UV–vis spectroscopy.

### 4.3. Sco^Sl^ possesses a redox active CXXXC motif but not thiol-disulphide reductase activity

**Figure 4.**
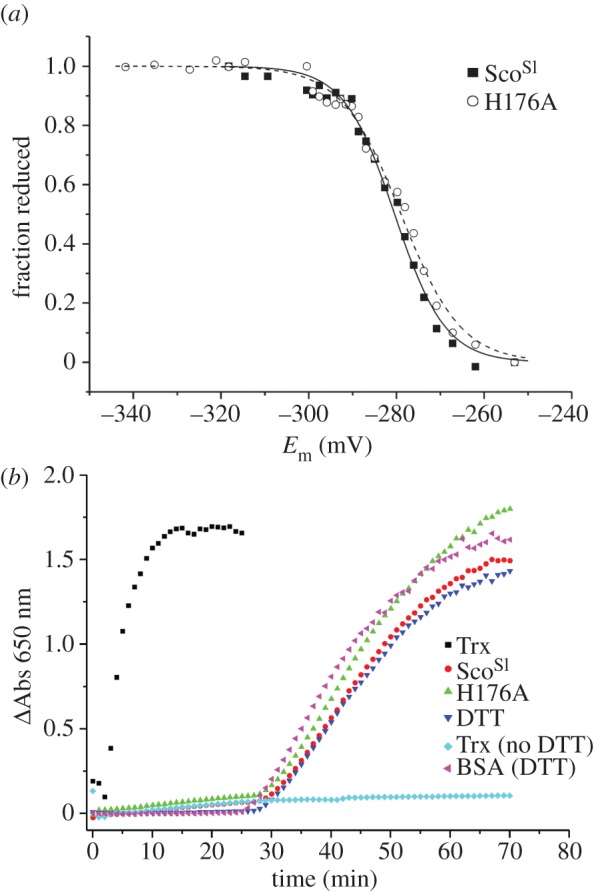
Mid-point redox potential and thiol-disulphide reductase activity of Sco^Sl^ and the H176A mutant. (*a*) Plot of fraction of reduced apo-Sco^Sl^ and the H176A mutant calculated from the fluorescence intensity at 330 nm as a function of the cell potential. The solid and dashed lines show the individual fits to the data for the respective proteins using equation (3.1). (*b*) Insulin reductase activity of *E. coli* Trx, Sco^Sl^ and the H176A mutant in the presence of 2.5 mM DTT, along with a number of controls, DTT only, BSA + DTT and Trx with no DTT. The precipitation of insulin was monitored at 650 nm. Sco^Sl^ or the H176A mutant do not show thioredoxin-like activity in this assay.

To explore a possible role for apo-Sco^Sl^ as a thiol-disulphide reductase, the mid-point redox potential (*E*_m_) of the Cys residues in the CXXXC motif of apo-Sco^Sl^ was first determined by measuring the difference in fluorescence intensity of W132 in the oxidized and reduced forms of apo-Sco^Sl^ upon addition of DTT (figure 4*a*). The data fit to the Nernst equation (3.1) to give an *E*_m_ value of –280 mV. An insulin precipitation assay with *E. coli* Trx as a control was used to assess whether Sco^Sl^ displayed thiol-disulphide reductase activity [40]. From the data presented in figure 4*b*, it is clear that Trx displays catalytic activity, whereas the long lag-phase followed by the slow increase in ΔA_650_ for apo-Sco^Sl^ is equivalent to the assay with DTT only and BSA with DTT (figure 4*b*), i.e. no catalytic activity. This indicates that despite having a redox active CXXXC motif, Sco^Sl^ does not possess catalytic thiol-disulphide reductase activity in the apo-form.

### 4.4. Sco^Sl^ is critical for morphological development and CcO activity at low exogenous Cu levels

**Figure 5.**
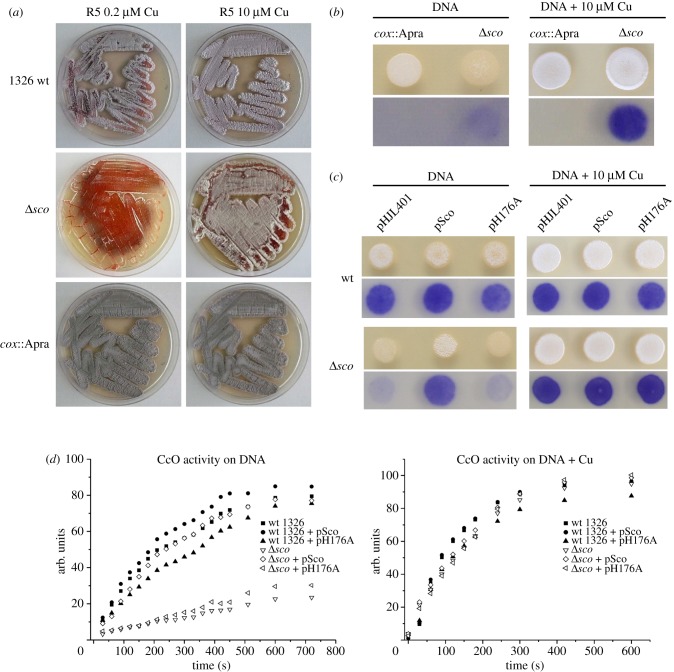
Morphology and CcO activity in wt and mutant strains of *S. lividans*. (*a*) *S. lividans* 1326 (wt), the Δ*sco* and the *cox*: :Apra mutants were grown on R5 and R5 supplemented with 10 μM Cu(II) for 5 days. (*b*) The top two frames show the growth of the *cox*::Apra and Δ*sco* mutants on DNA medium and DNA supplemented with 10 μM Cu(II). Drops of 10 μl containing 1000 spores were spotted on the plates and incubated for 48 h. The appearance of the white fluffy aerial mycelium and grey spores demonstrates that a strain is capable of full morphological development. The lower two frames show the *in vivo* detection of CcO activity with TMPD as substrate. The blue colour is the result of TMPD conversion to indophenol blue by CcO. (*c*) Morphology and CcO activity of wt and Δ*sco* mutant transformed with empty vector (pHJL401), pHJL401 containing *sco* under its own promoter (pSco) and pHJL401 expressing the H176A mutant (pH176A) on DNA plates and DNA supplemented with 10 μM Cu(II). (*d*) CcO activity detected by the TMPD assay and recorded by digital imaging of the plates. The ImageJ software (http://imagej.nih.gov/ij/) was used to calculate average pixel intensities (s.d. varied from 1% to 15%) of the indophenol blue stained mycelium as seen in (*b*) and (*c*). After correction for background reading, the data were multiplied by −1 and plotted as arbitrary units against time.

The function of Sco^Sl^ was probed by comparing morphological development and CcO activity of wt *S. lividans* 1326 and Δ*sco* strains. The wt strain shows almost full development, aerial hyphae and spore production on R5 medium, and a clear stimulation of development on R5 supplemented with 10 μM Cu(II) (figure 5*a*). The Δ*sco* mutant, on the other hand, is not capable of switching from vegetative to aerial growth and spore production on R5 medium (less than 0.2 μM Cu(II)), whereas development is clearly restored upon addition of 10 μM Cu(II) (figure 5*a*). This demonstrates that Sco^Sl^ has a critical role in the onset of aerial hyphae formation at low Cu availability. A similar effect on development of the Δ*sco* strain is seen on DNA medium under low levels of exogenous Cu(II) but development is once more restored on addition of 10 μM Cu(II) (figure 5*b*). A *cox*::Apra strain, in which the genes *SL2155* and *SL2156* encoding for subunits I and II of the *aa*_3_-type CcO have been removed, appears to be viable and not defective in development under low or elevated Cu(II) (figure 5*a,b*). However, development is somewhat delayed compared with the wt on both R5 and DNA medium. CcO activity was tested for in the *cox*::Apra mutant by using TMPD as substrate. It is apparent from figure 5*b* that no indophenol blue was observed and this is therefore consistent with the absence of TMPD oxidation by the *aa*_3_-type CcO, but also points to the absence of any other Cu/haem oxidases that may be upregulated in the absence of the *aa*_3_-type (the *bo*-type and *ba*_3_-type Cu/haem oxidases that do not react with TMPD are absent in *S. livdians*). In line with a putative function of Sco^Sl^ in the assembly of the Cu_A_ site, the CcO activity in the Δ*sco* mutant (figure 5*b*) is strongly reduced and maximal 25 per cent of the activity is found in the wt strain (figure 5*c,d*). Increasing the Cu(II) concentration in the medium clearly restores CcO activity to wt levels in the Δ*sco* mutant, corroborating the observation with development that Sco^Sl^ is also not required for maturation of CcO at elevated Cu(II) levels.

### 4.5. Sco^Sl^ binds Cu(II) rapidly in a biphasic process

**Figure 6.**
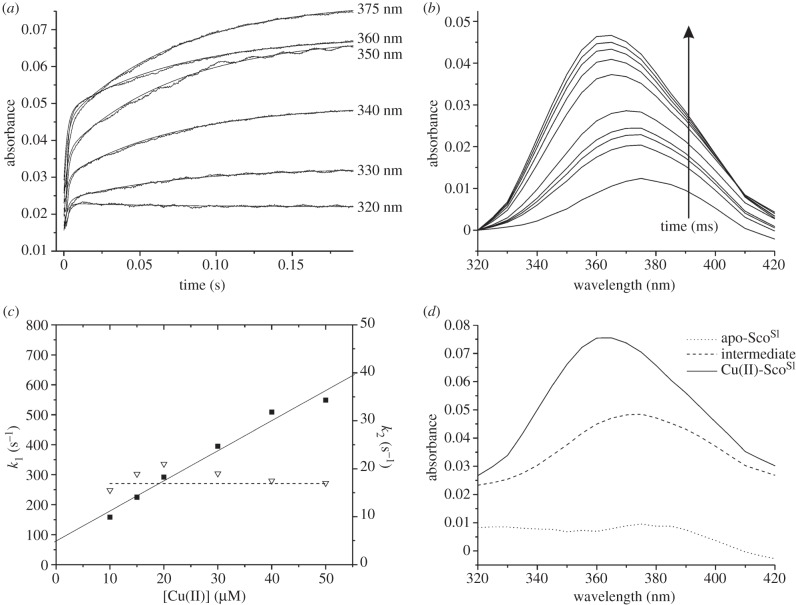
Stopped-flow kinetics of Cu(II) binding to Sco^Sl^. (*a*) Time courses at the indicated wavelengths observed on reacting 15 μM Sco^Sl^ with 50 μM Cu(II). (*b*) Spectra constructed at the following times, 3, 4.5, 6, 16.5, 60, 87, 114, 141, 200 ms, after mixing from the time courses given in (*a*). (*c*) First-order rate constants for the ‘fast’ (*k*_1_; filled squares) and ‘slow’ (*k*_2_; open triangles) phase of Cu(II) binding to Sco^Sl^ as a function of [Cu(II)]. The solid lines indicate a fit to the first rapid phase to obtain a second-order rate constant, and the dashed line indicates the independence of the rate of the slower phase (*k*_2_) on [Cu(II)]. (*d*) Spectra obtained from global fitting of the data presented in (*a*) and (*b*) to the model a > b > c.

On mixing reduced apo-Sco^Sl^ with excess Cu(II), a biphasic time course was observed at all wavelengths examined (figure 6*a*). The rapid phase occupied the first 10 ms after mixing and was followed by a slower phase taking approximately 200 ms to complete (figure 6*a*). This behaviour suggests that the formation of the final Cu(II)-Sco^Sl^ complex from the apo-protein passes through an intermediate. The time-dependent amplitudes of the time courses were used to construct the time-resolved spectra given in figure 6*b*. The observed rate constant for the faster process is seen to be linearly dependent on [Cu(II)] up to approximately 500 s^−1^. This phase therefore represents the second-order process in which Cu(II) binds initially to form an intermediate. Fitting of this dependency yields a value of the second-order rate constant (*k*_1_) of 1 × 10^7^ M^−1^ s^−1^. It is evident that at a [Cu(II)] of zero there is a significant value of the extrapolated pseudo first-order rate constant (79 s^−1^) (figure 6*c*). This value may be assigned to the dissociation rate constant (*k*_−1_) of Cu(II) from this intermediate. Taken together, these values allow an equilibrium binding constant (*K*_b_) of 1.3 × 10^5^ M^−1^ (*k*_1_/*k*_−1_) to be calculated for Cu(II) binding to the intermediate. A similar *K*_b_ value was determined from the examination of the amplitudes of the fast process as a function of [Cu(II)] (not shown). The slower kinetic process that leads from the intermediate to the final product showed little dependency on [Cu(II)], maintaining a value of approximately 20 s^−1^ at all [Cu(II)] used (figure 6*c*). This behaviour indicates that the step forming the final product from the intermediate involves no further Cu(II) binding but an internal rearrangement within the Cu(II) protein complex. Based on a sequential model with one intermediate, global fitting of the kinetic profiles in figure 6*a,b* gave rise to the spectral features of the intermediate and the final product as shown in figure 6*d*. It may be observed that the intermediate has a spectrum distinct from the final complex, having a *λ*_max_ at 375 nm and a considerably lower extinction coefficient (figure 6*d*). These spectral features are similar to those reported for the binding of Cu(II) to Sco^Bs^ [43]. The mechanism described earlier, namely a rapid second-order binding process leading to a spectrally distinct intermediate that then rearranges to form a final complex, is identical to that proposed for Sco^Bs^ [43] but in the case of Sco^Sl^ the rate constants are considerably larger.

### 4.6. H176 is a Cu(II) ligand and stabilizes the cupric state

**Figure 7.**
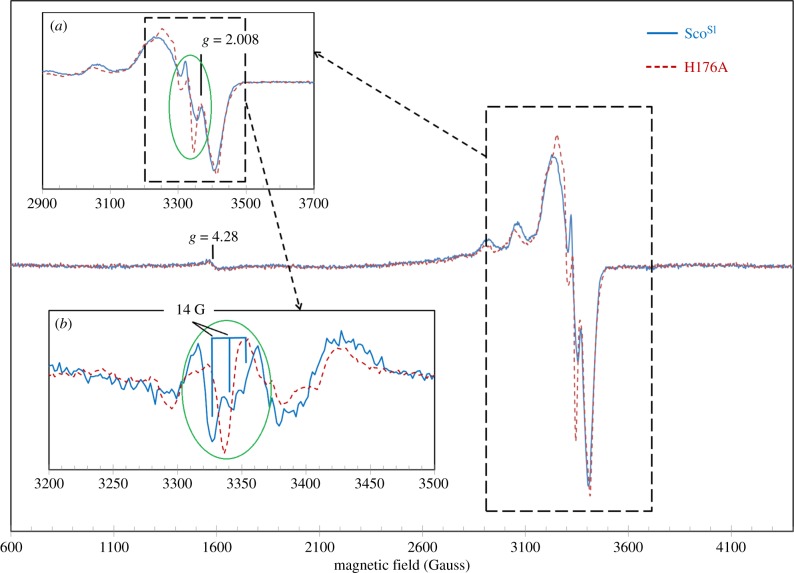
EPR spectra of Cu(II)-loaded Sco^Sl^ and the H176A mutant. The superimposed spectra within the dashed line box are shown in greater detail in inset (*a*). Derivatives by field have been taken of both spectra and inset (*b*) shows the second derivatives of the EPR absorption. The most rapidly changing part of the spectra, as the field increases, is highlighted by an oval in both first (inset *a*) and second derivatives (inset *b*) of the EPR absorption. The latter representation demonstrates that instead of a single peak in the H176A mutant, there is a triplet with a 14 G separation between the lines in the wt protein spectrum. Instrumental conditions were as follows: microwave frequency *ν*_MW_ = 9.466 GHz, microwave power *P*_MW_ = 3.181 mW, modulation frequency *ν*_m_ = 100 kHz, modulation amplitude *A*_m_ = 5 G, time constant *τ* = 81.9 ms, scan rate *V* = 22.6 G s^−1^, number of scans per spectrum NS = 1, each spectrum represents an average of three spectra obtained from three independent samples.

To explore further the mechanistic and functional properties of Cu(II)-Sco^Sl^, the effect of removing a putative first coordination sphere Cu(II) ligand was assessed by creating the H176A mutant. The EPR spectra of Cu(II)-loaded wt and H176A mutant are typical for cupric ions [44] (figure 7). The perfect fit of the baselines, including the *g* = 4.3 signal from the adventitious ferric iron in rhombic coordination [45], indicates that the differences in the EPR spectra of the two proteins, although small, are significant. An additional hyperfine structure is present in the wt spectrum (figure 7*a*, inset). This is better observed from the derivatives of the spectra (figure 7*b*, inset), showing that wt Sco^Sl^ has a triplet structure, whereas the mutant H176A has a single line. The separation of these three components in Gauss (approx. 14 G) is consistent with an interaction of the electronic spin with a nitrogen nucleus [46]. Thus, the differences in the EPR spectra of the two proteins can be explained by the presence of an additional N atom in the vicinity of the Cu(II) atom in the wt Sco^Sl^, whereas such an atom is absent in the H176A mutant, strongly suggesting that His176 is a Cu(II) ligand in Sco^Sl^.

**Figure 8.**
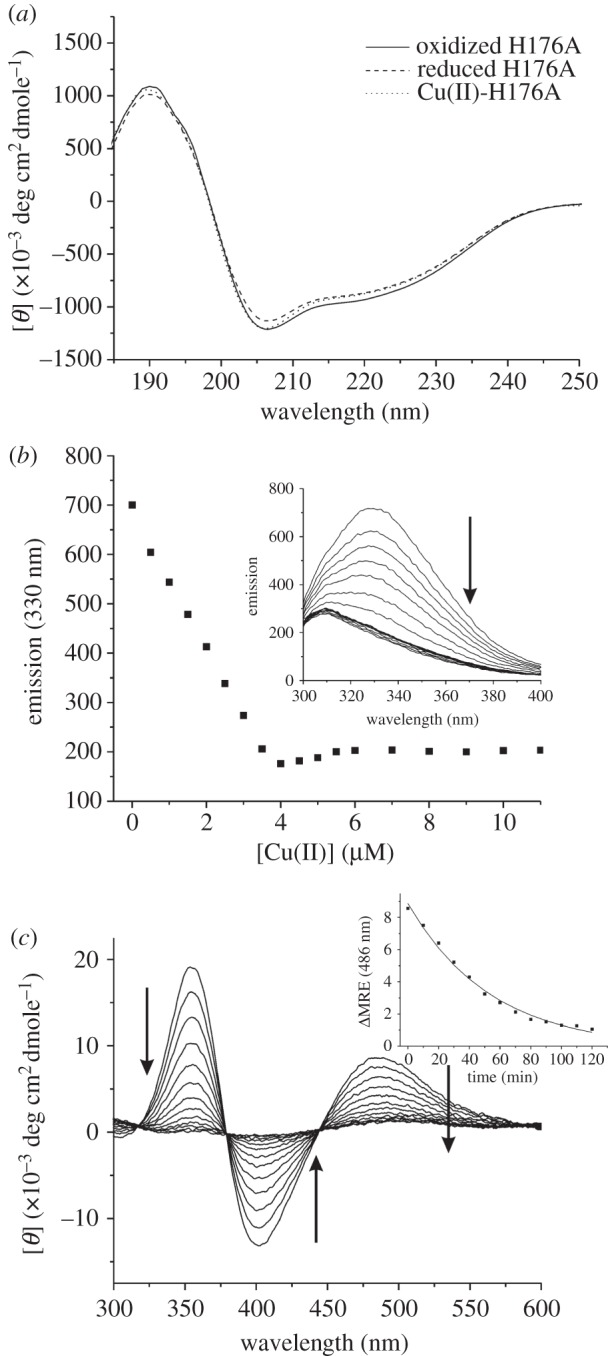
Cu(II) binding to the H176A mutant. (*a*) Far-UV CD spectra of apo-oxidized (disulphide), apo-reduced (free-thiol) and Cu(II)-loaded H176A. (*b*) Changes in the Trp emission spectrum of reduced apo-H176A (inset) upon titration with 0.5 μM of Cu(II)SO_4_ with the emission at 330 nm plotted as a function of [Cu(II)]/[H176A]. The arrow indicates the direction of the emission change up to 1 equivalent of Cu(II), with a slight increase and plateau observed above 1 equivalent of Cu(II). (*c*) Visible CD spectrum for the H176A mutant following addition of 1 equivalent of Cu(II). The spontaneous decay over time of the visible bands are indicated by arrows, and the inset is an example of a plot of mean residue ellipticity (MRE) at 486 nm versus time fitted to a single exponential decay function to give a decay rate constant (*k*) for the process. All spectra were recorded at 20°C, with protein concentrations ranging between 5 and 400 μM.

The far-UV CD spectra of the reduced and oxidized H176A Sco^Sl^ mutant are shown in figure 8*a* and the deconvolution using DichroWeb reported in table 1. The H176A mutant readily bound Cu(II) with slight shifts in *λ*_max_ compared with the wt protein (figure 2*b*). Stoichiometric Cu(II) binding was further corroborated from the decay of the W132 emission in the fluorescence spectrum (figure 8*b*) and the presence of a visible CD spectrum similar to that of wt Sco^Sl^, but with significant wavelength shifts of the peaks and trough (figure 3*b*). At Cu(II) stoichiometries of 1 or greater, the absorption bands in the UV–vis spectra spontaneously bleach, and a slight but significant increase in the W132 emission is observed (figure 8*b*). Similarly, the absorption bands in the visible CD spectrum spontaneously decay with time at Cu(II) stoichiometries of 1 or greater, with a decay rate, *k*_red_, of 3.1 × 10^−4^ s^−1^ (figure 8*c*, inset). The observation that the W132 emission does not return to apo-Sco^Sl^ intensities suggests that an autoreduction process is occurring resulting in Cu(I)-H176A. Finally, an *E*_m_ of −279 mV for the CXXXC motif in the H176A mutant was determined (figure 4*a*), and from the insulin precipitation assay catalytic thiol-disulphide reductase activity was absent for the apo-H176A (figure 4*b*).

### 4.7. Complementation of the Δ*sco* strain with pSco^Sl^ restores function but pH176A does not

To test the effect of the H176A mutant on development and CcO activity, complementation experiments with low copy number plasmids expressing the wt Sco^Sl^ gene (pSco) and the H176A mutant (pH176A) were carried out. For transformation of pSco and pH176A into the 1326 wt strain, no effect on growth or development on DNA at low [Cu] or with 10 μM Cu(II) was observed. However, a small and reproducible effect on CcO activity was observed on DNA without added Cu(II). Figure 5*d* illustrates that the H176A mutation has a negative effect on CcO activity in the wt strain, whereas the wt Sco protein has a small positive effect. In the Δ*sco* mutant, the development and CcO activity under low Cu(II) concentrations are restored to near wt 1326 levels upon transformation with pSco (figure 5*c*,*d*). However, pH176A was not capable of restoring development or CcO activity to wt levels in the Δ*sco* mutant although a small increase in activity is observed in the absence of exogenous Cu(II) (figure 5*d*). We also note that the time courses for the TMPD assays are multiphasic in the absence of added Cu(II). At 10 μM Cu(II), the development and CcO activity in all Δ*sco* transformants on DNA are restored to wt levels (figure 5*c,d*), and the TMPD time courses now concur to a single phase (figure 5*d*). Taken together, these data indicate that the phenotype and reduced CcO activity in the Δ*sco* mutant can be attributed completely to the absence of Sco^Sl^, and that despite the H176A mutant being able to stoichiometrically bind Cu(II) *in vitro*, it is not capable of supporting wt levels of development and CcO activity at low Cu(II) concentrations *in vivo*.

### 4.8. The H176A mutant reveals a spectrally distinct intermediate on binding Cu(II)

**Figure 9.**
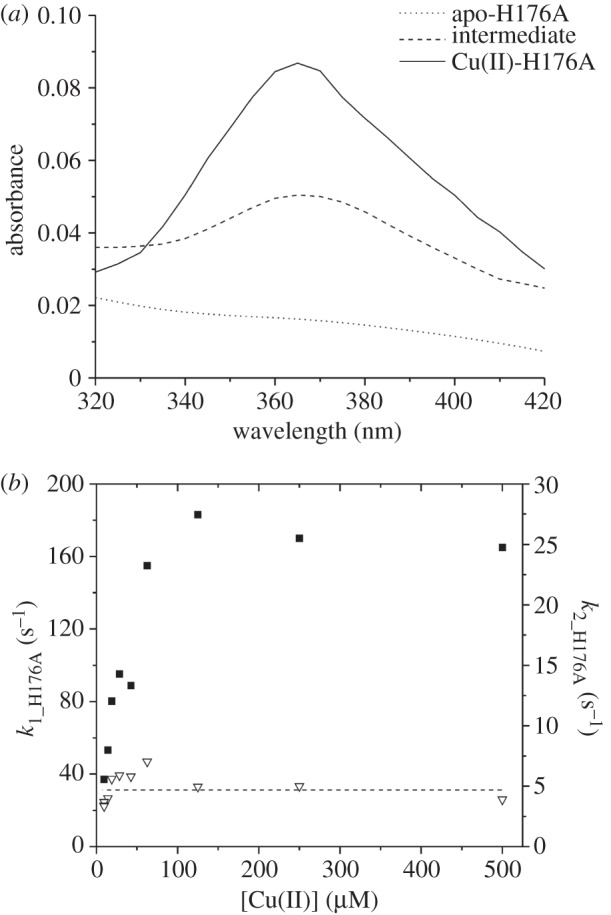
Stopped-flow kinetics of Cu(II) binding to the H176A mutant. (*a*) Global fitting of time courses at wavelengths from 420 to 320 nm to a sequential model a > b > c. (*b*) First-order rate constants for the ‘fast’ (*k*_1_H176A_; filled squares) and ‘slow’ (*k*_2_H176A_; open triangles) phases of Cu(II) binding to the H176A as a function of Cu(II) concentration. The dashed line indicates the [Cu(II)] independence for *k*_2_H176A_.

The kinetics of Cu(II) binding to the H176A mutant were studied in a similar manner to those of the wt Sco^Sl^ and revealed rapid formation of an intermediate that eventually yielded the final complex. Similar analysis to that undertaken for the wt protein shows that the intermediate formed in the H176A mutant is distinctly different in spectral characteristics from that shown in figure 6*d*, with a *λ*_max_ now at 365 nm (figure 9*a*). The initial rapid kinetic process again displayed a Cu(II) concentration dependence but now the pseudo first-order rate constant plateaued at approximately 170 s^−1^ (figure 9*b*). Fitting of the initial linear portion of the rate dependence at low [Cu(II)] yields a second-order rate constant (*k*_1_H176A_) of 2 × 10^6^ M^−1^ s^−1^ and a dissociation rate constant (*k*_−1_H176A_) of 30 s^−1^. Thus, the rates of binding and dissociation appear lower than in wt Sco^Sl^ and correspond to a slightly lower *K*_b_ for the intermediate complex of 6.7 × 10^4^ M^−1^. The slow process again shows very little dependence on [Cu(II)], yielding a first-order rate constant (*k*_2_H176A_) of approximately 5 s^−1^, again significantly slower than wt.

## 5. Discussion

### 5.1. The *sco* operon of *Streptomyces lividans* encodes a gene for a Cu(II) binding Sco protein

Previous studies with Cu(II)-Sco proteins from human, yeast and *B. subtilis* [16,20,23,42,47] have identified a type 2 Cu centre, with UV–vis absorption spectra displaying similar features to the red cupredoxin nitrosocyanin that binds Cu(II) in a square pyramidal geometry using two N(His) ligands, a single S(Cys), a solvent water molecule in the basal plane and a coordinating O(Glu) as the axial ligand [48]. The major absorption band in the visible region for Cu(II)-nitrosocyanin (approx. 390 nm) and Cu(II)-Sco (approx. 360 nm) are reported to arise from S(Cys)–Cu(II) charge transfer dominated by σ rather than π orbital interactions as is the case in cupredoxins [49]. The absorption spectra for Cu(II)-Sco^Sl^ and the H176A mutant (figure 2*b*) have the same overall features as Cu(II)-Sco^Bs^ [23], the Cu(II)-H135A mutant of Sco^Bs^ [50] and the eukaryotic Sco1 proteins form yeast and human [20,47], corroborating that the *3966* gene, part of the *sco* operon in *S. lividans*, encodes for a Cu(II) binding Sco protein. From the EPR data (figure 7), a contribution to the Cu(II) coordination sphere from the imidazolate N of His176 is apparent, yet the H176A mutant retains the ability to bind Cu(II) with the same stoichiometry and a similar affinity (derived from kinetic studies) as the wt protein. However, in the absence of the His ligand, the Cu(II)-Sco^Sl^ is susceptible to autoreduction to Cu(I). This has also been reported for the H135A mutant of Sco^Bs^ but occurs an order of magnitude faster (1 × 10^−3^ s^−1^) compared with the H176A Sco^Sl^ mutant (3.1 × 10^−4^ s^−1^) [50].

### 5.2. Sco^Sl^ is unlikely to function as a thiol-disulphide reductase

An alternative ‘non-Cu’ role for bacterial Sco proteins as thiol-disulphide reductases has been postulated, based on the ability of Sco^Tt^ to reduce the Cys residues of the Cu_A_ site in CcO, thus facilitating Cu delivery by the periplasmic Cu(I) chaperone PCu_A_C [18]. To maintain Sco^Tt^ in the reduced state competent for redox function, a supply of reducing equivalents in the periplasmic environment is required and thus a redox role has been questioned [51]. Although we have not looked at the same reaction with the *S. lividans* proteins, namely the reduction of the Cys residues in the Cu_A_ domain of CcO by Sco^Sl^, our *in vitro* assay clearly shows no enzymatic enhancement of thiol-disulphide reductase activity for apo-Sco^Sl^ compared with *E. coli* Trx (figure 4*b*). Our findings are thus in keeping with results from a similar assay with human Sco1, where thiol-disulphide activity was not detected [20]. An *E*_m_ of −280 mV for the CXXXC motif of Sco^Sl^ has been determined in this study and is comparable to that of the *E*_m_ for the CXXC motif of Trx (−270 mV). However, these similarities in *E*_m_ are not sufficient to infer catalytic activity in apo-Sco^Sl^ when present in an insulin precipitation assay where Trx clearly possesses thiol-disulphide activity and, therefore, catalytic activity is not inherent on the *E*_m_ of the disulphide motif in the respective proteins. Furthermore, the *E*_m_ of the H176A mutant is unaffected, and this finding coupled with the observation that Cu(I) is the more stable oxidation state of the Cu bound H176A has ramifications for interpreting the lack of development and CcO activity *in vivo* with this mutant (vide infra).

### 5.3. At low Cu levels morphogenesis and CcO activity require the assistance of Cu(II)-Sco^Sl^

The absence of indophenol blue from the TMPD assay in the *cox*::Apra strain is consistent with no activity from the *aa_3_*-type oxidase (figure 5*b*). However, in the Δ*sco* mutant indophenol blue is detected, albeit to a much lower level than in the wt strain or upon addition of exogenous Cu(II) (figure 5*b–d*). Therefore, a basal level of active CcO is present under low Cu and in the absence of Sco^Sl^, suggesting that the Cu_A_ site of CcO is metallated and active. Despite the presence of low levels of CcO activity in the Δ*sco* mutant, the developmental switch from vegetative to aerial mycelium clearly does not occur in the absence of Sco^Sl^ (figure 5*a*). The morphological phenotype for the Δ*sco* mutant coinciding with the significantly decreased CcO activity compared with the wt strain is restored upon complementation with pSco (figure 5*c,d*). This is therefore evidence that the switch from vegetative to aerial growth in *S. lividans* is dependent on Sco^Sl^ with a clear correlation to CcO biogenesis implied. It therefore appears that when Sco^Sl^ is present CcO is metallated much more efficiently at low [Cu]. In contrast at elevated [Cu], the development block is lifted, and CcO activity is restored in the Δ*sco* mutant, indicating that under these conditions the role of Sco^Sl^ is bypassed with Cu being delivered to the Cu_A_ site through another means or by spontaneous self-assembly.

The H176A mutant binds Cu(II) readily, but is susceptible to an autoreduction process that results in the Cu(I)-bound state. The inability of the H176A mutant to restore CcO activity in the Δ*sco* mutant under low [Cu(II)] may therefore be due to the mutant's inability to maintain the Cu(II) oxidation state, with the Cu(I) bound state no longer active in transferring Cu to the Cu_A_ site. The absence of CcO activity and development with this mutant further questions the role of the proximal *3965* gene that encodes for a PCu_A_C-like protein in the co-factoring of the Cu_A_ domain. Recent data from *R. sphaeroides* are in accordance with our data supporting the maturation of the Cu_A_ site proceeding only via a Sco-like protein, PrrC, with a PCu_A_C-like protein having an as yet undefined role [52]. Although not tested in this study, it is conceivable that Cu plays a role in thiol-disulphide redox activity of Sco^Sl^ and that the stabilization of the cuprous state by the H176A mutant inhibits any electron donation involving Cu oxidation. Further studies into this role are planned.

### 5.4. A second Cu(II)-Sco^Sl^ target is required for aerial hyphae formation in *Streptomyces lividans*

As indicated, despite the presence of low levels of CcO activity in the Δ*sco* mutant, the developmental switch from vegetative to aerial mycelium clearly does not occur in the absence of Sco^Sl^ (figure 5*a*). If deleting the *sco* gene would be equivalent to only strongly reducing the CcO activity, the phenotypes of the Δ*sco* and *cox*::Apra mutants would be expected to be very similar. However, this is not the case as the *cox*::Apra mutant is still capable of full development at low [Cu(II)], whereas the Δ*sco* mutant is stalled in the vegetative growth phase (figure 5*a*). This leads us to speculate that a second Sco^Sl^ target is involved in initiation of aerial hyphae formation. This Cu-Sco^Sl^ target is likely to be a cuproenzyme and its identification is currently in progress. Furthermore, the observation that a strain lacking the terminal *aa*_3_-type oxidase can survive and initiate a full life cycle demonstrates that another terminal oxidase, incapable of reacting with TMPD, can take over respiration (figure 5*b*). Genes for the terminal quinol *bd*-type oxidase are present in *S. lividans* and is thus a good candidate.

### 5.5. Sco^Sl^ captures exogenous Cu(II) rapidly

**Figure 10.**
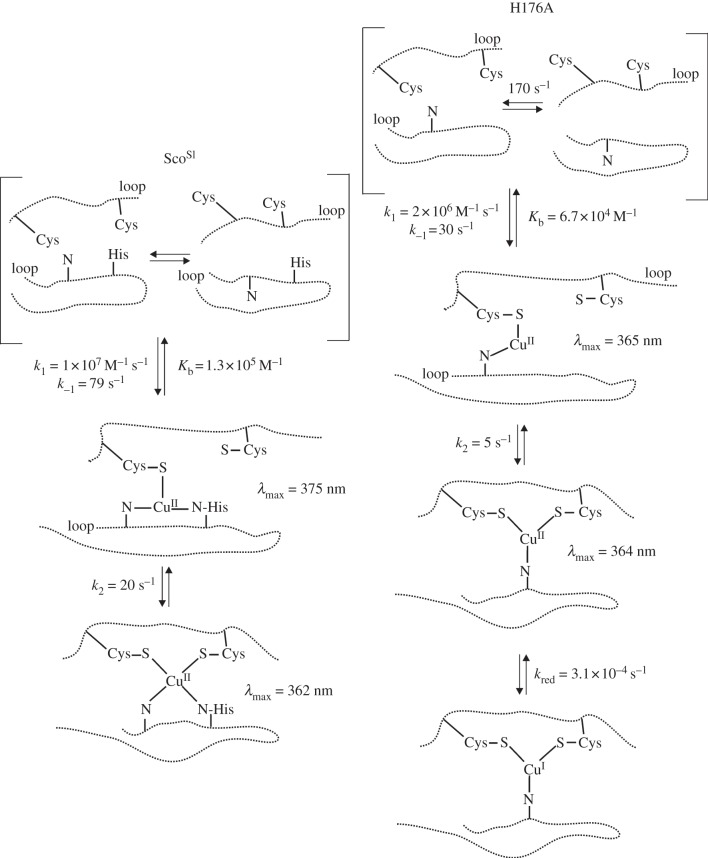
Mechanistic features of Cu(II) capture and binding to Sco^Sl^ and the H176A mutant.

While our data establish that Sco^Sl^ acts as a Cu(II)-metallochaperone, how it acquires Cu(II) *in vivo* is not known. Sco^Sl^ is exported via the secretory (Sec)-pathway so is likely to obtain Cu once folded in the extracellular environment through a ligand-exchange interaction with another Cu-chaperone [51]. Under low Cu levels, it is expected that the cells' most efficient chaperone and scavenging systems operate, facilitating the efficient delivery of Cu to required locations, consistent with our *in vivo* data for Sco^Sl^ at low [Cu]. An emerging paradigm is that periplasmic (and possibly extracellular) cuproproteins may obtain Cu not from periplasmic or extracellular pools but from Cu that has been routed via the cytosol and delivered by P_1_-type ATPases [53]. Regardless of how Sco^Sl^ is co-factored *in vivo*, the mechanism through which Cu(II) binds to apo-Sco^Sl^ *in vitro* may best be understood through figure 10. The loops containing the Cu binding Cys residues and the His residue in Sco proteins (figure 1*b*) have been reported to be highly dynamic in the absence of Cu [22]. Therefore, the apo-Sco^Sl^ can be considered to have multiple conformations in equilibrium with one another, with some possessing a pre-formed Cu(II) binding site (figure 10). Rapid freeze EPR experiments with Sco^Bs^ have indicated prior to Cu(II)-thiolate coordination an initial capture complex exists composed of an equatorial N-Cu(II)-N complex, likely involving H135 and a N atom, possibly from a backbone amide [54]. By including this information in our model, we envisage that an initial capture complex comes close to one of the thiols and forms a site to which Cu(II) binds in a second-order process to form an intermediate in which the Cu(II) is coordinated to one thiol, His176 and a backbone amide N with a *λ*_max_ = 375 nm (figure 10). This spectroscopically observable intermediate is consistent with absorption spectra reported for the single Cu(II)-thiolate coordinating mutants, C45A and C49A, in Sco^Bs^ [42] where a *λ*_max_ of approximately 380 nm is observed, assigned to coordination of Cu(II) by a single Cys, His-135 and two unknown O/N ligands [42]. The protein dynamics in the apo-form is very rapid compared with Cu(II) binding to the pre-formed site and thus the spectral change we observe, dominated by binding to the thiol, is second-order. Once formed, the intermediate undergoes rearrangement in which the Cu(II)-complex is presented to the second thiol ligand leading to the final Cu(II)-Sco^Sl^ complex with a twofold increase in absorption and a *λ*_max_ shift to 362 nm. This rearrangement occurs with *k*_2_ approximately 20 s^−1^ and a rate constant for dissociation (*k*_−2_) based on experiments with high concentrations of Cu(II) chelators of less than 10^−6^ s^−1^ (data not shown). While the binding of Cu(II) to form the intermediate is relatively weak (*K*_b_ = 10^5^ M^−1^), coupling to the rearrangement that has an estimated equilibrium constant in favour of the final complex of 10^7^ (*k*_2_/*k*_−2_) yields an overall affinity for Cu(II) greater than 10^12^ M^−1^. Although it appears that Cu(II) capture undergoes a similar mechanism to that reported for Sco^Bs^, the rates for Sco^Sl^ are considerably quicker (*k*_1_ > three orders of magnitude, *k*_2_ > 10 times faster) indicating that Sco^Sl^ is much more efficient in Cu(II) capture and rearrangement to the final complex. Our experiments were carried out under similar conditions (temperature, pH and ionic strength) and thus structural differences inherent between proteins or the dynamics of the loops housing the Cys and His residues governing the accessibility to Cu(II) may be a reason.

### 5.6. H176 plays a significant role in Cu(II) capture

The second-order rate constant for the H176A mutant is significantly lower than wt (figure 10) and probably reflects the differences in the geometry of the intermediate site in the absence of the His residue. The spectrum of the intermediate is again dominated by thiol binding but the spectral modulation (position of *λ*_max_) given by His coordination is absent in the mutant compared with wt (figure 10). Interestingly, the His coordination appears to modulate the spectrum of the intermediate to a greater extent than the final complex in which both thiols bind to the Cu(II) (figure 10). The second-order nature of Cu(II) binding gives way to a first-order process at high [Cu(II)] (figure 9*b*), indicating that the rate limit for the formation of the intermediate species becomes rate limited by the rate at which the binding site can be formed in the absence of H176. This may be unremarkable given the different stereochemistries required to bring a His-N into the site as opposed to an amide-N close to the thiol. The intermediate nevertheless rearranges, albeit on a slower time scale than wt, to form the final product, in keeping with the kinetic restraints imposed by the absence of the His. The absence of H176 therefore does not impede Cu(II) capture but in its absence it becomes less efficient. H176 therefore clearly has a role in Cu(II) capture and is critical for maintaining the cupric form that is essential for the functional role of Sco^Sl^.

## 6. Acknowledgement

This study was supported by the University of Essex through the award of a PhD studentship to K.L.I.M.B.
